# Advances in Aquaporins

**DOI:** 10.3390/cells12020303

**Published:** 2023-01-13

**Authors:** Giuseppe Calamita

**Affiliations:** Department of Biosciences, Biotechnologies and Environment, University of Bari Aldo Moro, 70125 Bari, Italy; giuseppe.calamita@uniba.it; Tel.: +39-0805442928

Aquaporins (AQPs) are a family of transmembrane channel proteins, widespread throughout nature, where they facilitate the diffusion of water and small solutes into and out of both cells and organelles [1,2]. They are widely studied in living organisms owing to the different roles they play—some of them even unexpected. Aquaporins are also implicated in several clinical disorders and their interest to the field of medicine is strong.

This Special Issue addresses many aspects of the fascinating family of AQP membrane channels, including their functions and regulation in biology and medicine, biophysical properties, relevance as biomarkers, evolutionary pathways, and translational value in pharmacology and biotechnologies. The Special Issue represents a valuable basis for current and future research. The content of the papers that make up the Special Issue is briefly summarized in the paragraphs below.

AQP4 is the major aquaporin in the central nervous system where it is expressed to non-neuronal glial cells, mainly to astrocytes. AQP4 is particularly abundant in the membrane of astrocyte processes, which align brain capillaries and pia. Murine and human astrocytes express some of the reported isoforms of AQP4 which are believed to underlie the plasma membrane assemblies of astrocytes, the so-called orthogonal arrays of particles (OAPs). OAPs have been suggested to exert various roles in astrocyte and CNS tissue physiology. The paper by Jorgačevski et al. reviews the cellular distribution of several AQP4 isoforms and their functional involvement in OAP assembly, a process which is regulated by various intracellular and extracellular proteins [3].

AQP4 is of critical importance in brain water and volume homeostasis, its involvement reported across a large spectrum of pathological disorders. Growing evidence suggests a proinflammatory function of AQP4 in the astrocytic cytokines release, with activation of microglia and other astrocytes. Roles for astrocyte AQP4 have been shown in the neuroinflammation associated to Parkinson’s disease (PD). In their paper, using the Parkinsogenic toxin MPP+ and *Aqp4* knockout mice, Prydz at al. provide further evidence regarding the proinflammatory function of AQP4 in PD, which is suggested to be secondary to the dysregulation of astrocytic volume homeostasis [4].

Using a rat model of status epilepticus, a condition of prolonged seizure activity, Kim and coworkers show that a blockade of 67-kDa laminin receptor facilitates AQP4 downregulation and blood–brain barrier disruption through the activation of ERK1/2- and p38 MAPK-Mediated PI3K/AKT [5]. This is consistent with previous studies, with rodents showing downregulation of piriform cortex AQP4 in vasogenic edema formation. The authors suggest that 67LR-p38 MAPK/ERK1/2-PI3K-AKT-AQP4 signaling cascades may underlie both serum extravasation and the expression of AQP4 in astroglio-vascular systems, providing useful information for novel therapeutic targets to treat the vasogenic edema that accompany several neurological disorders.

Septic shock is the most severe complication of sepsis, leading to systemic inflammation following bacterial infection. This entails multiple organ failure leading to a dramatically elevated mortality. A strong interest has been triggered by AQP9, an AQP channel of broad selectivity primarily expressed in hepatocytes and leukocytes, as a potential target to treat septic shock-related mortality. In their work, using both a cellular model and a murine model of LPS-induced endotoxemia, Tesse and coworkers find a role for AQP9 in the early acute phase of LPS-induced endotoxic shock involving NF-κB signaling [6]. This finding is in line with other studies, where AQP9 was associated with inflammatory and infectious responses [7,8].

By means of a prospective validation study, Bergmann and coll. provide evidence of major adverse kidney events associated with the AQP5 -1364A/C promoter polymorphism in sepsis [9]. The work in question confirms the functional importance of the AQP5 -1364A/C single nucleotide promoter polymorphism in altering key mechanisms of inflammation and survival in sepsis by increasing the risk of acute kidney injury. The AQP5 -1364A/C polymorphism is also suggested to be an independent prognostic factor in sepsis.

Graft rejection and cytomegalovirus infection are major complications of post-transplantation kidneys related to T-cell function, which depends on the AQP3 expression level. In their work with adult patients within 12 months after renal transplantation, Rump and coll. explored the impact of the AQP3 A(−1431)G promoter polymorphism on kidney transplant patients [10]. The AQP3 A(−1431)G A-allele was seen to confer more resistance against cytomegalovirus infection and to be associated with enhanced immune cell migration and AQP3 expression in T-cells. This outlines the importance of the genotype-specific modulation of AQP3 expression in the management of immunosuppression and antiviral prophylaxis after kidney transplantation.

In their review, Gao and coworkers give an updated overview of the genetic and molecular mechanisms of congenital nephrogenic diabetes insipidus, a rare hereditary disease [11]. A specific focus is place on the potential disease-causing mutations in the antidiuretic hormone receptor 2 (AVPR2) and AQP2, the molecular aberrations in the AVPR2 and AQP2 mutants, post-translational alterations in terms of phosphorylation, glycosylation, and ubiquitination, and various protein–protein interactions that modulate tetramerization, phosphorylation, trafficking, stability, ubiquitination, and degradation of AQP2. The review is particularly useful for those who work on the pathophysiology and pharmacology of NDI.

AQP11, a superaquaporin, is an endoplasmic reticulum (ER)-resident protein that transports water, glycerol, and hydrogen peroxide. AQP11 has been suggested to be involved in ER stress induced by lipotoxicity and inflammation in human obesity. The study by Frühbeck and coworkers, conducted with morbidly obese patients with and normal-weight individuals shows that proinflammatory factors such as TNF-α, and particularly TGF-β1, downregulate AQP11 at the mRNA and protein levels while increasing its subcellular distribution surrounding lipid droplets [12]. Importantly, the AQP11 gene knockdown is found to increase the basal and TGF-β1-induced expression of the ER markers ATF4 and CHOP. Owing to its peroxiporin feature, AQP11 overexpression in visceral fat is suggested to represent a compensatory mechanism aimed at ameliorating ER stress in obesity.

The major role of salivary glands is the production and secretion of saliva, a function for which AQPs exert the key role in ensuring the osmotic efflux of water out of the acinar cells. In their review, D’Agostino and coll. provide a summary of the expression, localization, roles and modulation of AQPs in adult human, rat and mouse salivary glands [13]. The authors address the involvement of AQPs in clinical forms leading to salivary hypofunction and consequent xerostomia, such as in Sjögren’s syndrome. Diabetes, agedness, head and neck cancer radiotherapy and salivary gland cancer are also reviewed, and the potential value of AQP5 as therapeutic target in strategies for the treatment of xerostomia is evaluated.

Water homeostasis is crucial in various reproductive processes such as oocyte transport, hormonal secretion, fertilization, blastocyst formation, pregnancy, and birth, and the AQP-mediated water transport is reported to play important roles. AQPs are also implicated in spermatogenesis and spermatozoa storage [14]. In their review, Kordowitzki and coll. provide an update on the physiology of AQPs in the female reproductive system and on their relevance in reproductive disorders and aging [15]. 

Several reports suggest that AQPs may act as regulators of mesenchymal stem cell (MSC) proliferation owing to their rapid cell volume regulation. Crucial roles have also been invoked for AQPs in modulating MSC attachment to the extracellular matrix, their spread, and migration. An original review by Zannetti and coworkers discusses the relevance of AQPs in MSC physiology and their potential translational importance in regenerative and reparative medicine [16].

Increases in AQP expression are associated with greater severity of many cancers, in particular, in augmenting migration and invasiveness, as occurs in glioblastoma and colon cancers. Khan and coll. provide a timely review of published work, indicating that AQPs exert distinct roles in cancer metastasis, angiogenesis, and resistance to apoptosis [17]. The pharmacological relevance of AQPs targeting for use as potential drug targets to reduce female and male reproductive cancer cell growth and invasiveness is elaborated.

Assessing the relevant mode of action is vital for developing new drugs and predicting potential mechanisms of resistance. Sensitivity of African trypanosomes to melarsoprol and pentamidine is mainly mediated by TbAQP2, an aquaglyceroporin expressed by trypanosomes. In their elegant paper, Petersen and Beitz show that the ionophores carbonyl cyanide m-chlorophenyl hydrazone (CCCP) and Gramicidin (but not Nigericin) inhibit *Trypanosoma brucei* aquaglyceroporins TbAQP2 and TbAQP3 at neutral pH [18]. The study recommends caution when working with ionophores, suggesting suitable controls to rule out unwanted ionophore side effects, even at concentrations used to disturb the transmembrane ion distribution.

Transmembrane glycerol transport is an ancient biophysical property that evolved in selected groups of AQP water channel proteins. In their fascinating study, Yilmaz and coworkers conduct broad-level genome and transcriptome analyses to shed light onto the duplication history of aquaglyceroporins in Deuterostomia [19]. Tandem duplication is found to be the main mechanism of gene expansion in echinoderms and hemichordates, which, together with whole-genome duplications in the chordate lineage, continued to shape the genomic repertoires in craniates. The paper unravels the origins and diversification of aquaglyceroporins which are over 800 million years old, providing novel bases for proposing a pandeuterostome aquaglyceroporin gene nomenclature.
